# Prognostic Value of Tumour-Infiltrating Lymphocytes in an Unselected Cohort of Breast Cancer Patients

**DOI:** 10.3390/diagnostics12102527

**Published:** 2022-10-18

**Authors:** Kathleen Schüler, Daniel Bethmann, Sandy Kaufhold, Carolin Hartung, Kathrin Stückrath, Tilmann Lantzsch, Christoph Uleer, Volker Hanf, Susanne Peschel, Jutta John, Marleen Pöhler, Jörg Buchmann, Karl-Friedrich Bürrig, Edith Weigert, Christoph Thomssen, Eva Johanna Kantelhardt, Martina Vetter

**Affiliations:** 1 Department of Gynaecology, Martin Luther University Halle-Wittenberg, 06120 Halle (Saale), Germany; 2 Institute of Pathology, Martin Luther University Halle-Wittenberg, 06120 Halle (Saale), Germany; 3 Department of Gynaecology, Hospital St. Elisabeth and St. Barbara, 06110 Halle (Saale), Germany; 4 Gynäkologisch-Onkologische Praxis, 31134 Hildesheim, Germany; 5 Department of Gynaecology, Nathanstift, Hospital Fürth, 90766 Fürth, Germany; 6 Department of Gynaecology, St. Bernward Hospital, 31134 Hildesheim, Germany; 7 Department of Gynaecology, Helios Hospital Hildesheim, 31135 Hildesheim, Germany; 8 Department of Gynaecology, Asklepios Hospital Goslar, 38642 Goslar, Germany; 9 Institute of Pathology, Hospital Martha-Maria, 81479 Halle (Saale), Germany; 10 Institute of Pathology Hildesheim, 31135 Hildesheim, Germany; 11 Institute of Pathology, Hospital Fürth, 90766 Fürth, Germany; 12 Institute of Epidemiology, Biometry and Informatics, Martin Luther University Halle-Wittenberg, 06108 Halle (Saale), Germany

**Keywords:** tumor infiltrating lymphocytes, early breast cancer, prognosis, cohort study

## Abstract

Tumour-infiltrating lymphocytes (TILs) are considered to have prognostic and predictive value for patients with early breast cancer. We examined 1166 breast cancer patients from a prospective, multicentre cohort (Prognostic Assessment in Routine Application (PiA), *n* = 1270, NCT 01592825) following recommendations from the International TILs Working Group. TIL quantification was performed using predefined groups and as a continuous variable in 10% increments. The primary objective was the distribution of TILs in different breast cancer types. The second objective was the association with the recurrence-free interval (RFI) and overall survival (OS). Stromal infiltration with more than 60% TILs appeared in 2% of hormone receptor (HR)-positive and HER2-negative tumours, in 9.8% of HER2-positive tumours (any HR) and 19.4% of triple-negative breast cancers (TNBCs). Each 10% increment was associated with an improvement in the prognosis in HER2-positive samples (RFI, hazard ratio 0.773, 95% CI 0.587–1.017; OS, hazard ratio 0.700, 95% CI 0.523–0.937). When defining exploratory cut-offs for TILs, the use of a 30% threshold for the HR-positive and HER2-negative group, a 20% threshold for the HER2 group and a 60% threshold for the TNBC group appeared to be the most suitable. TILs bore prognostic value, especially in HER2-positive breast cancer. For clinical use, additional research on the components of immune infiltration might be reasonable.

## 1. Introduction

Breast carcinoma (BC) is a heterogeneous disease and, in addition to established clinical and histopathological parameters, tumour-infiltrating lymphocytes (TILs) are discussed as additional biomarkers that predict clinical outcomes. Knowing that tumour development and progression are influenced by the microenvironment of the tumour tissue consisting of, e.g., cancer cells, cancer-associated fibroblasts, endothelial cells, nerves, vasculature and lymphocytes [1], TILs have emerged as a reproducible biomarker for BC groups (see Dieci et al. for a current review) [2,3]. The impact of lymphocyte stromal infiltration in breast cancer and a strategy for the evaluation of these TILs were defined as standardised factors in tumour tissue in the recommendations of the International TILs Working Group for assessing TILs in breast cancer several years ago [4]. TILs were shown to be an independent prognostic factor for TNBC, as a 10% TIL increase is associated with a 17% risk reduction for recurrence or death. For HER2-positive tumours, the studies are more controversial, but TILs may help to predict benefits from anti-HER2 therapy or T-cell checkpoint inhibitors (see review [5,6]).

## 2. Materials and Methods

Our prospective biomarker study was designed and reported according to the REMARK recommendations (Reporting Recommendations for Tumour Marker) [7], using an unselected cohort of early BC patients (*n* = 1270) that were enrolled from five certified German breast cancer centres from 2009 to 2011 (PiA study—Prognostic Assessment in Routine Application, NCT 01592825). An observation time of five years with a median follow-up time of 63.3 months (1–132) was available.

Patients were included at the time of diagnosis if the following inclusion criteria were met: female patients aged 18 years or older with early invasive, non-metastasised breast cancer and no other malignancy. There was no limitation regarding tumour size; lymph node status; grading; or receptor status, e.g., oestrogen receptor (ER), progesterone receptor (PgR) and human epidermal growth factor receptor 2 (HER2). The tumour staging was performed in accordance with the UICC TNM rules [8] and the grading was performed according to Elston and Ellis [9]. The receptor statuses for ER, PgR and HER2 were assessed by the local pathologist of the centres using FFPE tissue from surgical excisions or core needle biopsies before any treatment. Tumours were considered HR-positive if more than 1% of the tumour cells expressed ER or PgR, or if the samples had an immune reactivity score of at least 3 [10]. The HER2 status was defined on the basis of the DAKO-score; samples were considered positive with a DAKO-score of 3, and in situ hybridisation was performed in the case of a DAKO-score of 2 [11]. Patients were treated in accordance with the annually updated German Guidelines (AGO) at the time of enrolment [12]. From the total cohort of 1270 patients, 1070 underwent primary surgery or neoadjuvant chemotherapy (NACT) (*n* = 200).

### 2.1. TIL Evaluation

TILs were assessed using haematoxylin and eosin (HE)-stained FFPE sections of tumour tissue before any treatment according to the recommendations of the International TILs Working Group [4]. TILs were recorded depending on the percentage of stromal area covered by mononuclear immune cells, determining the average TIL proportion for the entire tumour area. Only lymphocytes inside the borders of the invasive tumour, including the invasive margin, were evaluated; immune infiltration outside the tumour border, necrosis and tertiary lymphoid structures were excluded.

**Figure 1:**
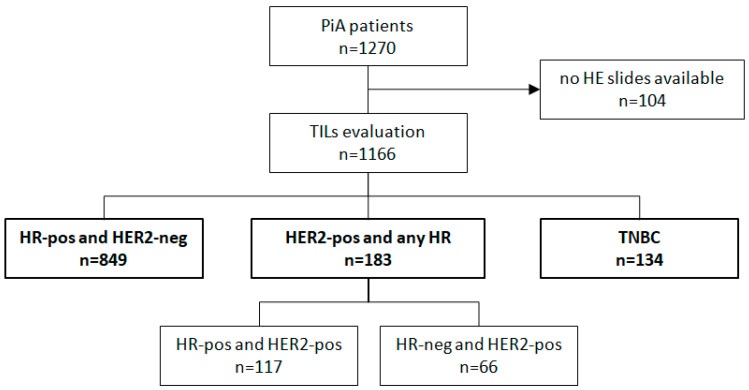
CONSORT diagram: patients with TIL evaluations (*n* = 1166) and in the subgroups according to HR and HER2 expressions. Abbreviations: hormone receptor (HR), human epidermal growth factor receptor 2 (HER2), triple-negative breast cancer (TNBC).

HE-stained slides were available for 1166 of the 1270 tumours (91.8%, Figure 1). Since core biopsies were taken outside of the participating centres, more HE slides were missing from NACT patients compared with tumour tissue from patients with the primary surgery (34% versus 3.4%). We grouped samples into three predefined IHC types by considering the steroid hormone receptor and HER2. HE slides were available for 93.6% of the HR-positive and HER2-negative group (*n* = 849), 86.7% of the HER2-positive group irrespective of the HR status (*n* = 183) and 88.2% of TNBC group (*n* = 134) (Supplementary Table S1).

The median age of the TILs cohort was 61 years, but patients with HER2-positive tumours and TNBCs were younger (median age 56 years) than the HR-positive and HER2-negative patients (median age 62 years), the tumours were larger and less differentiated and the patients had more lymph node involvement than patients with an HR-positive status (Table 1). In 36 cases (3.1%), patients in the TILs cohort did not receive the recommended therapy due to patient choice or frailty.

Two independent TIL researchers were blinded to any diagnostic and clinical data. In the case of inconsistencies, the slides were observed together and subsequently reevaluated. TILs were scored as a continuous parameter in 10% increments and additionally assigned to one of these three TIL groups: low TILs (<10%), intermediate TILs (10–60%) and high TILs (>60%). The 10% cut-off was chosen to form a group with almost no TILs in the stroma; the 60% threshold was chosen to characterise a group with strong lymphocyte infiltration, as proposed by Denkert C. et al. in 2015 [13]. We performed an exploratory univariate analysis using the Cox model to define the most suitable cut-offs for our TILs cohort.

### 2.2. Outcome Assessment and Statistical Analysis

The primary objective of this study was to investigate the distribution of stromal TILs in the predefined and clinically relevant immunohistochemical (IHC) groups. The secondary objectives were the association of TILs with the recurrence-free interval (RFI) and overall survival (OS) in univariate and multivariate analyses. The endpoints were determined according to the standardised definitions for efficacy endpoint (STEEP) criteria [14]. The RFI was defined as the interval from diagnosis until any event, including local recurrence, distant recurrence and death from breast cancer and OS was defined as the time from diagnosis until death from any cause. For univariate analysis of the three TILs groups, we used Kaplan–Meier estimates and differences were described using a log-rank test. The univariate and multivariate proportional hazard regression models were used to estimate the impact of a 10% TIL increase and the dichotomous parameters.

Our findings in the multivariate analysis in the different IHC groups were visualised as forest plots (Review Manager Version 5.3) using 10% TIL increments. A recursive partitioning analysis (classification and regression tree (CART)) was performed to define homogeneous risk groups with regard to the potential prognostic impact of TILs [15,16] using a cut-off of 20%.

To estimate the association between TILs and therapy success in patients who had been treated with neoadjuvant chemotherapy (NACT), we used the pathological complete response (pCR) with the strict definition with no cancer cells in the breast or axilla (ypT0, ypN0) [17].

The clinical and histopathological parameters of the TILs study group were assessed using the chi-squared test. All *p*-values were two-sided and those less than 0.05 were considered significant. We performed all these analyses using SPSS 25 (IMB, Armonk, NY, USA).

## 3. Results

### 3.1. TIL Evaluation in Different Histopathological Types

**Figure 2:**
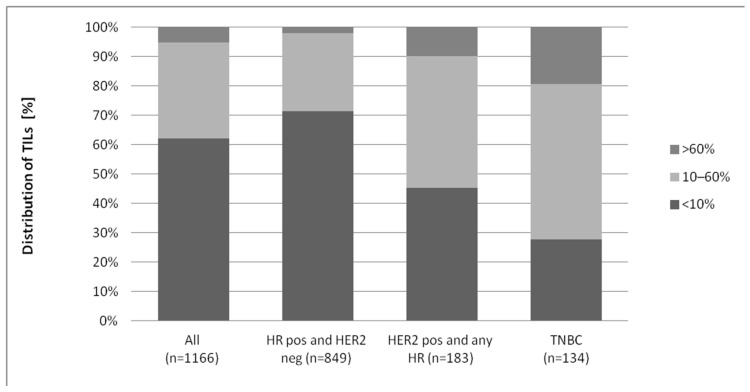
Distributions of TILs (*n* = 1166).

Almost two third of the tumours showed less than 10% TILs in the stroma and 5% showed more than 60% TILs. Considering the different IHC types, a distinct distribution of TILs was detected. In HR-positive HER2-negative tumours, almost three quarters showed less than 10% TILs, whereas HER2-positive tumours and TNBCs showed higher amounts of TILs (55.4% and 72.2%, respectively, with more than 10% TILs) (see Figure 2).

### 3.2. TILs and Association with Clinical Outcome

We found a weak association between TILs and survival in the entire TILs cohort (*n* = 1166); when applying 10% TIL increments, the multivariate analysis revealed a hazard ratio of 0.92 (95% CI 0.821–1.026) for the RFI and a hazard ratio of 0.92 (95% CI 0.826–1.023) for the OS (Supplementary Table S2). The impact of TILs when considering the RFI and OS was different in the three IHC groups (summarised in Table 2). In the HR-positive group (*n* = 849), patients with low and intermediate TILs infiltration (<60%) had a similar course of disease; in contrast, patients with 60% and more TILs had the worst probability regarding their RFI and OS in this group (Supplementary Figure S1A,B). In the context of stepwise 10% TIL increments and the cut-off analysis, no impact of TILs was observed (Table 2).

A notable effect of TILs was detected for the HER2-positive group (irrespective of the HR status, *n* = 183). All 18 patients with more than 60% TILs were free of any recurrence; nine events occurred in the intermediate TILs group (88.4, 95% CI 81.3–95.5), while the TILs group with less than 10% showed the worst outcome (81.9, 95% CI 73.3–90.5). A significant impact of TILs was seen for the OS, with only 71.6% (95% CI 61.4–81.8) of the patients having less than 10% TILs and 90.8% (95% CI 84.3–97.3) of the intermediate TILs group and 94.4% (95% CI 83.8–≥99.9) of the high TILs group achieving a five-year survival (Supplementary Figure S1C,D). We detected a lower risk of recurrence with each 10% TILs increment (hazard ratio 0.76, 95% CI 0.586–0.993, *p* = 0.044); furthermore, after an adjustment for tumour size and grading, there was a 22.7% reduction per 10% TILs increment (hazard ratio 0.77, 95% CI 0.587–1.017, *p* = 0.066). Additionally, a 10% TILs increment significantly predicted a 30% reduction in the risk of death in the adjusted analysis (see Table 2).

As expected, patients with TNBC (*n* = 134) had the most unfavourable prognosis of all BC patients, with 73.5% (95% CI 65.9–81.1) of the TNBC patients being recurrence-free after five years and 76.7% being still alive (95% CI 69.4–84.0). Patients with less than 10% TILs had the worst outcome, as 65.6% (95% CI 49.5–81.7) were free of RFI events compared with 72.1% (65% CI 61.3–82.9) and 88.5% (95% CI 76.2–≥99.9) of the intermediate and high TILs groups, respectively (Supplement Figure S1E,F). The same effect of TILs was seen for OS: 67.6% (95% CI 52.5–82.7) probability for less than 10% TILs, 77.7% (95% CI 67.7–87.7) for 10–60% TILs and 88.5% (95% CI 76.2–≥99.9) for more than 60% TILs. Considering the 10% increase in the multivariate analysis, we observed a 9.7% reduced risk for any disease-specific event and a 6.0% reduction for OS (Table 2).

**Figure 3:**
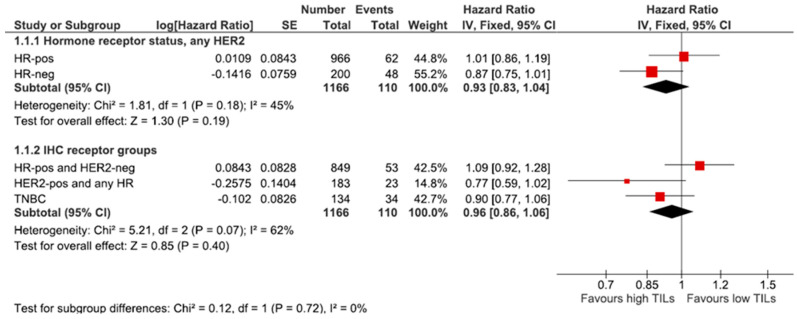
Forest plot for the effect of TILs infiltration per 10% increment in different receptor groups regarding the RFI. Red box: point estimate of the effect for a single group, diamond: overall effect estimate of all groups.

TILs showed a greater prognostic impact in the HR-negative group than in the HR-positive group (Figure 3). For patients with HR-negative and node-negative tumours, we found a significantly improved recurrence-free interval via a CART analysis using 20% TILs as a cut-off for 9% of our study patients (*n* = 100), as shown in Supplementary Figure S2.

### 3.3. TILs and Association with pCR

TILs analysis was available for 132 of the 200 neoadjuvant-treated patients. Overall, neoadjuvant therapy resulted in a pCR rate of 26.5%. Due to the small number of cases with NACT in the different IHC types, further association analyses were not feasible.

### 3.4. TILs and Cut-Off Analyses

**Figure 4:**
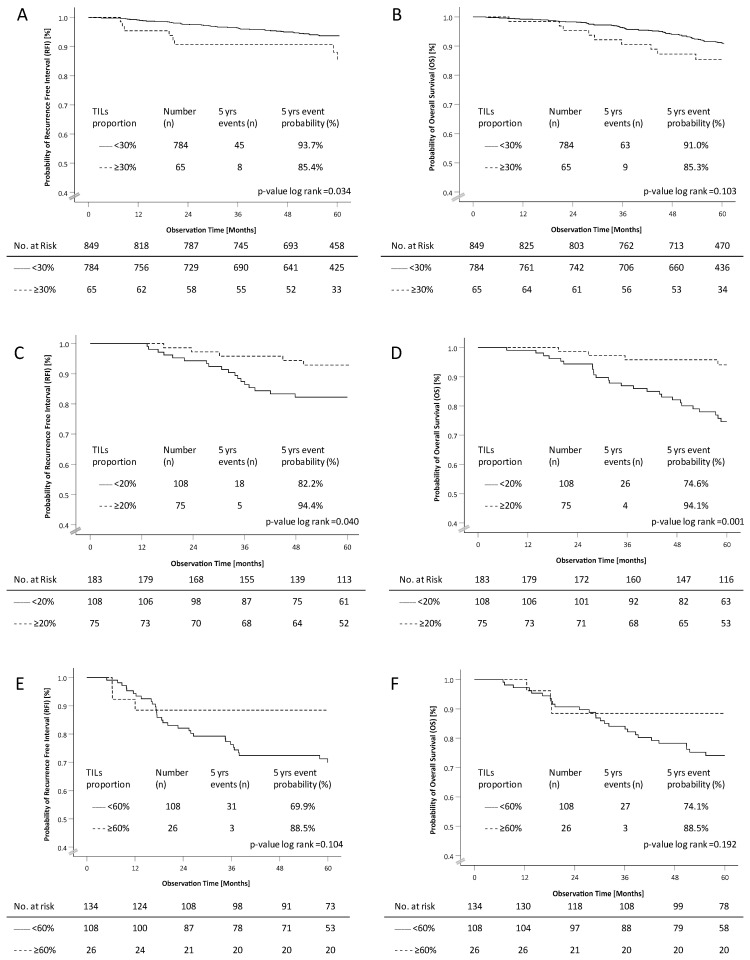
Kaplan–Meier plots for patients in the TILs groups with their cut-offs for the HR-pos and HER2-neg RFI (**A**) and OS (**B**), HER2-pos and any HR RFI (**C**) and OS (**D**), and TNBC RFI (**E**) and OS (**F**); the tables present the effective sample size for each interval (no. at risk).

We tried to estimate the optimal cut-off value of TILs with regard to the RFI in the total cohort and the different IHC groups using the maximum likelihood method in an exploratory univariate analysis (see Supplementary Table S3). The most suitable threshold for risk assessment in the HR-positive group was found at 30%, demonstrating that patients with more than 30% TILs had a significantly worse RFI than those with 30% or less (hazard ratio 2.21, 95% CI 1.041–4.683) (Figure 4A,B; Table 2). No difference in outcome (RFI) between patients who received chemotherapy and patients who did not receive chemotherapy was observed (hazard ratio 2.04 vs. 2.09 respectively). In the HER2-positive group, the optimal cut-off was estimated at 20%, resulting in an improved RFI for patients with a high TIL infiltration (hazard ratio 2.71, 95% CI 1.004–7.290) and also an improved OS (hazard ratio 4.81, 95% CI 1.689–13.799) (Figure 4C,D). The effect was also detectable for patients who had received adjuvant trastuzumab (*n* = 164, RFI hazard ratio 3.00, 95% CI 1.001–8.962; OS hazard ratio 7.72, 95% CI 1.811–32.940). Using 60% TILs as a suitable threshold for dichotomous assessment regarding TNBC, patients had a 2.5-fold (95% CI 0.789–8.453) higher risk for RFI events and a 2.1-fold (95% CI 0.658–7.156) higher risk for death with a lower infiltration of TILs (<60%; Figure 4E,F). Again, the effect was independent of chemotherapy treatment. For the adjusted hazard ratio of TILs cut-offs with other clinical and pathological factors, see Supplementary Table S4.

## 4. Discussion

In our study (*n* = 1166), we investigated the distribution of TILs overall and in prespecified and clinically relevant subgroups, as well as their association with the course of the disease. According to the international guidelines, we focussed on the description of stromal TILs.

With regard to the distribution of TILs, our results were in line with published data, which shows that TILs are more likely in HER2-positive tumours, and the highest amount of TILs are found in TNBC [18]. These tumours with a high risk of recurrence seem to have more immunogenic characteristics and subsequently gain from higher amounts of TILs [3].

For our patients with HER2-positive tumours, a 10% increment in TILs corresponded to a better prognosis. In a pooled analysis of NACT trials, Denkert and colleagues found a significantly longer disease-free survival in the HER2-positive group for tumours with high TILs [13], which was also shown by Heppner et al. [19] and Loi et al. [20]. In addition, dichotomous discrimination revealed a survival benefit for HER2 patients with 20% or more TILs, as was previously seen in the retrospective analysis of the ShortHER trial, where the same cut-off was used [21]. In summary, the amount of TILs was confirmed as a prognostic factor in this group.

In the heterogeneous group of TNBC patients, our results were comparable with the findings of the above-mentioned pooled analysis (hazard ratio 0.92, 95% CI 0.87–0.98, DFS) [13], as well as the meta-analysis of Gao and colleagues, who calculated a pooled hazard ratio of 0.93 for DFS (95% CI 0.90–0.96) from ten retrospective single studies. Focussing only on TNBC, Adams and colleagues showed an association between a 10% TILs increase and survival when considering metastasis and death from breast cancer [22]. In addition to published data, the benefit of high infiltration in HER2-positive breast cancer and TNBC manifested after two years of observation in our cohort, while in the first few years, other factors seemed to have a more dominant influence on event-free survival.

Corresponding to the low amounts of TILs, HR-positive and HER2-negative tumours generated a low immune response. Previous data on HR-positive tumours is contrary. The negative effect of immune infiltration in luminal breast cancer was described by Denkert et al. [13], whereas Lundgren et al. described a positive effect of TILs infiltration after 30 years of observation [23].

We showed no association with survival in our cohort regarding the 10% increment. This result was also seen in other studies, as summarized in the meta-analysis of He et al. [24]. In contrast to that, using a 30% cut-off, the prognostic effect of TILs was demonstrated in our study.

Our analysis results lead to the suggestion of detecting patients with low amounts of TILs in HER2-positive and TN cancers, as they form a group with an especially high risk of recurrences.

Our decision to use three groups of TIL proportions to determine the impact of TILs was found to be unsuitable for survival analysis and, as a result, not applicable in a clinical context. Up to now, no suitable cut-off has been recommended or implemented in the guidelines, as the definition of lymphocyte-predominant breast cancer even varies between studies. For routine clinical practise, not only recommendations for assessing TILs but also for their precise application are needed.

Given the uncertainty of the clinical use and the inconsistent results in different studies, there are many ideas for improving TILs evaluation. Characterisation of the immune infiltration by gene expression analysis might be one way, e.g., Kochi and colleagues analysed the genomic signatures of 22 TIL-associated genes and deduced their ability to predict clinical outcomes and the effect on the prognosis. A significant association between immune genes and survival benefit was again only detectable in HER2-positive tumours. Regarding the hypothesis of high amounts of TILs leading to a better outcome, in most studies without significant results, TILs alone were not shown to be a reliable biomarker. The distinction of different infiltrating immune cells could lead to more explicit results. In 2015, Salgado and colleagues mentioned in their recommendations about TILs evaluation that immune infiltration could be divided into tumour-suppressing cells, e.g., Th 1 cells and NK cells, as well as cells that support tumour progression, such as tumour-promoting macrophages and Tregs [4]. Moreover, the therapeutic consequence of interactions between tumour cells and immune cells, such as the PD1 and PD-L1 signalling pathway and their relation to TILs, were examined [25].

In contrast to other costly and time-consuming techniques, TILs evaluation is a cheap and easily determined marker that is evaluated using haematoxylin and eosin (HE) slides and has potential prognostic value in a clinical context that still needs to be improved for reliable use.

## 5. Conclusions

Using these analyses of a prospective patient cohort, it was demonstrated that TILs infiltration differed in the IHC groups of breast cancer patients. The feasibility of TILs evaluation and its prognostic impact of TILs was presented by working with data from a clinical cohort. Our study showed that the cut-offs must be calculated separately for the IHC groups of breast cancer patients, with special attention given to patients with HER2-positive tumours.

## Figures and Tables

**Table 1:** Patients’ characteristics and histopathological parameters regarding TIL evaluations of the tumours at the time of diagnosis.

Parameters	HR-Pos and HER2-Neg, *n* = 849			HER2-Pos and Any HR, *n* = 183			TNBC, *n* = 134		
	<10%	10–60%	>60%	<10%	10–60%	>60%	<10%	10–60%	>60%
	*n* = 605	*n* = 227	*n* = 17	*n* = 83	*n* = 82	*n* = 18	*n* = 37	*n* = 71	*n* = 26
	*n* (%)	*n* (%)	*n* (%)	*n* (%)	*n* (%)	*n* (%)	*n* (%)	*n* (%)	*n* (%)
**Age**									
≤50 years	118 (60.8) **	**70 (36.1)**	**6 (3.1)**	24 (38.1)	32 (50.8)	7 (11.1)	14 (29.2)	24 (50.0)	10 (20.8)
>50 years	**487 (74.4)**	**157 (24.0)**	**11 (1.7)**	59 (49.2)	50 (41.7)	11 (9.2)	23 (26.7)	47 (54.7)	16 (18.6)
**Tumour size**									
<2 cm	335 (68.9)	141 (29.0)	10 (2.1)	26 (35.6)	37 (50.7)	10 (13.7)	7 (15.9) *	**22 (50.0)**	**15 (34.1)**
≥2 cm	270 (74.4)	86 (23.7)	7 (1.9)	57 (51.8)	45 (40.9)	8 (7.3)	**30 (33.3)**	**49 (54.4)**	**11 (12.2)**
**Nodal status**									
Negative	393 (71.6)	146 (26.6)	10 (1.8)	53 (50.5)	43 (41.0)	9 (8.6)	18 (26.5)	37 (54.4)	13 (19.1)
Positive	212 (70.7)	81 (27.0)	7 (2.3)	30 (38.5)	39 (50.0)	9 (11.5)	19 (28.8)	34 (51.5)	13 (19.7)
**Tumour differentiation**									
G1	115 (74.7) *	**37 (24.0)**	**2 (1.3)**	7 (87.5)	1 (12.5)	0 (0.0)	0 (0.0) *	**1 (100.0)**	**0 (0.0)**
G2	**416 (72.6)**	**147 (25.7)**	**10 (1.7)**	48 (46.6)	45 (43.7)	10 (9.7)	**24 (42.9)**	**25 (44.6)**	**7 (12.5)**
G3	**74 (60.7)**	**43 (35.2)**	**5 (4.1)**	28 (38.9)	36 (50.0)	8 (11.1)	**13 (16.9)**	**45 (58.4)**	**19 (24.7)**

* *p*-value (Pearson χ^2^ test) < 0.05, ** *p*-value (Pearson χ^2^ test) < 0.005. Abbreviations: hormone receptor (HR), human epidermal growth factor receptor 2 (HER2), triple-negative breast cancer (TNBC).

**Table 2:** Multivariate analyses of TILs considering the recurrence-free interval (RFI) and overall survival (OS).

		Multivariate Analyses of RFI			Multivariate Analyses of OS		
		Hazard Ratio	95% CI	*p*-Value	Hazard Ratio	95% CI	*p*-Value
**TILS per 10% increment**							
HR-pos and HER2-neg		1.088	0.925–1.280	0.310	1.062	0.918–1.229	0.416
HER2-pos and any HR		0.773	0.587–1.017	0.066	0.700	0.523–0.937	0.016
TNBC		0.903	0.768–1.063	0.220	0.94	0.794–1.113	0.476
**TILs cut-offs**							
HR-pos and HER2-neg	≥30% vs <30%	1.834	0.833–4.038	0.132	1.374	0.666–2.835	0.390
HER2-pos and any HR	<20% vs ≥20%	2.469	0.892–6.837	0.082	3.877	1.339–11.226	0.012
TNBC	<60% vs ≥60%	2.034	0.601–6.878	0.254	1.645	0.485–5.582	0.424

Abbreviations: hormone receptor (HR), human epidermal growth factor receptor 2 (HER2), triple-negative breast cancer (TNBC).

## Data Availability

The data generated in this study are available within the article and its supplementary data files. Raw data were generated and processed from the authors and are available on request to the corresponding authors.

## Supplementary Materials

The following supporting information can be downloaded from https://www.mdpi.com/article/10.3390/diagnostics12102527/s1. Table S1: Patients’ characteristics and histopathological parameters of the tumours (based on local pathology) used for TILs analyses. Table S2: Multivariate analysis of the entire TILs cohort (*n* = 1166). Table S3: Cut-off exploration for TILs in the three receptor groups. Table S4: Multivariate analyses of TILs and adjusted factors by considering the recurrence-free interval (RFI) and overall survival (OS). Figure S1: Kaplan–Meier plots for patients in the TILs groups with their cut-offs for the HR-pos and HER2-neg RFI (**A**) and OS (**B**), HER2-pos and any HR RFI (**C**) and OS (**D**), and TNBC RFI (**E**) and OS (**F**); the tables present the effective sample size for each interval (no. at risk). Figure S2: Classification and regression tree (CART) for TILs as a dichotomous parameter (cut-off of 20%).
