# Supplementary material for: Prognostic Value of Tumour-Infiltrating Lymphocytes in an Unselected Cohort of Breast Cancer Patients
Source: Diagnostics (Basel). 2022 Oct 18;12(10):2527. doi: 10.3390/diagnostics12102527 (PMC9601161; doi:10.3390/diagnostics12102527)
Supplement: Supplementary file 1 [file diagnostics-12-02527-s001.zip › diagnostics-1935374-supplementary .pptx]

## Slide 1
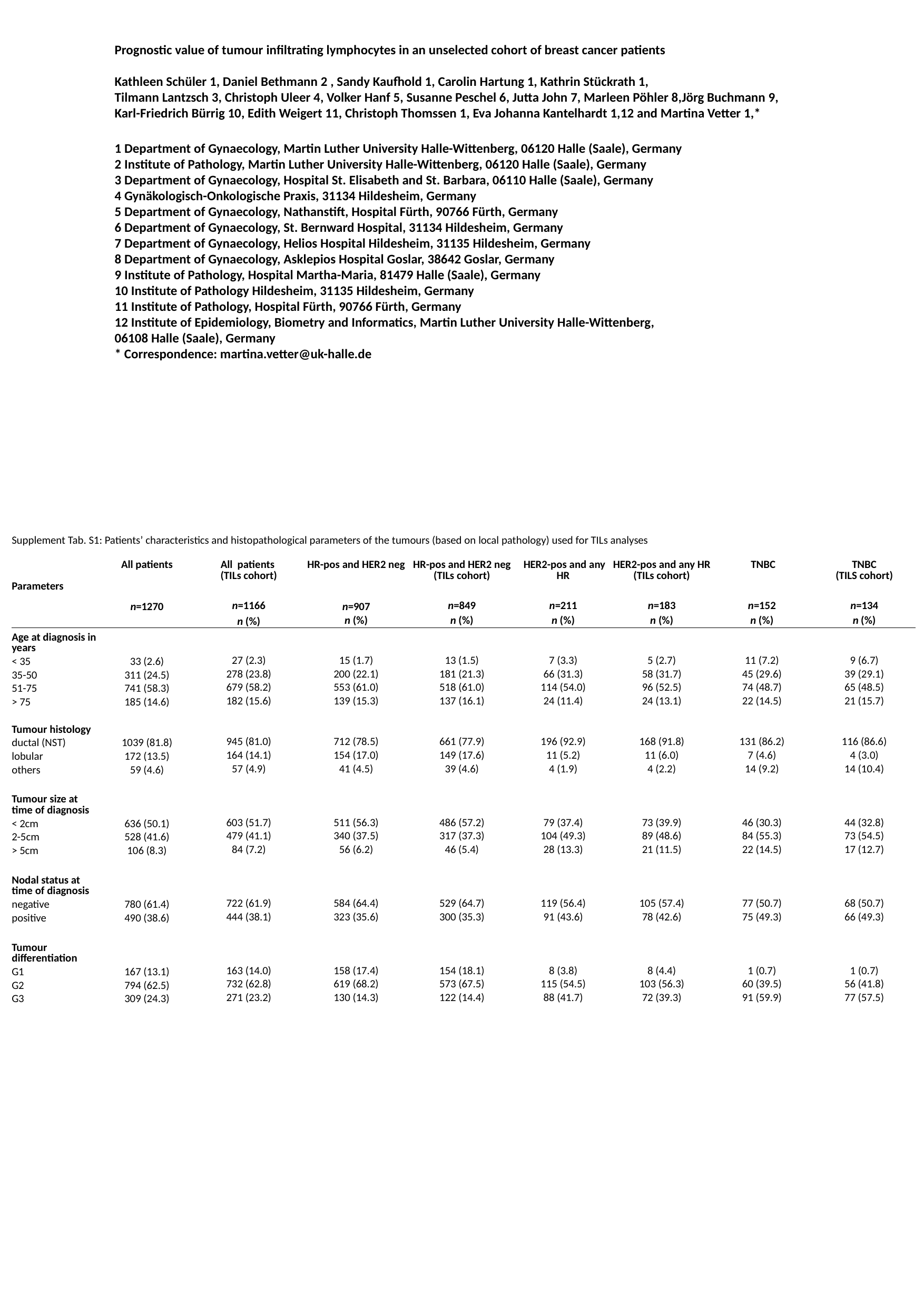

Prognostic value of tumour infiltrating lymphocytes in an unselected cohort of breast cancer patients
Kathleen Schüler 1, Daniel Bethmann 2 , Sandy Kaufhold 1, Carolin Hartung 1, Kathrin Stückrath 1,
Tilmann Lantzsch 3, Christoph Uleer 4, Volker Hanf 5, Susanne Peschel 6, Jutta John 7, Marleen Pöhler 8,Jörg Buchmann 9, Karl-Friedrich Bürrig 10, Edith Weigert 11, Christoph Thomssen 1, Eva Johanna Kantelhardt 1,12 and Martina Vetter 1,*
1 Department of Gynaecology, Martin Luther University Halle-Wittenberg, 06120 Halle (Saale), Germany
2 Institute of Pathology, Martin Luther University Halle-Wittenberg, 06120 Halle (Saale), Germany
3 Department of Gynaecology, Hospital St. Elisabeth and St. Barbara, 06110 Halle (Saale), Germany
4 Gynäkologisch-Onkologische Praxis, 31134 Hildesheim, Germany
5 Department of Gynaecology, Nathanstift, Hospital Fürth, 90766 Fürth, Germany
6 Department of Gynaecology, St. Bernward Hospital, 31134 Hildesheim, Germany
7 Department of Gynaecology, Helios Hospital Hildesheim, 31135 Hildesheim, Germany
8 Department of Gynaecology, Asklepios Hospital Goslar, 38642 Goslar, Germany
9 Institute of Pathology, Hospital Martha-Maria, 81479 Halle (Saale), Germany
10 Institute of Pathology Hildesheim, 31135 Hildesheim, Germany
11 Institute of Pathology, Hospital Fürth, 90766 Fürth, Germany
12 Institute of Epidemiology, Biometry and Informatics, Martin Luther University Halle-Wittenberg,
06108 Halle (Saale), Germany
* Correspondence: martina.vetter@uk-halle.de
| Supplement Tab. S1: Patients’ characteristics and histopathological parameters of the tumours (based on local pathology) used for TILs analyses |
| --- |
| Parameters | All patients | All patients (TILs cohort) | HR-pos and HER2 neg | HR-pos and HER2 neg (TILs cohort) | HER2-pos and any HR | HER2-pos and any HR (TILs cohort) | TNBC | TNBC (TILS cohort) |
| --- | --- | --- | --- | --- | --- | --- | --- | --- |
| | n=1270 | n=1166 | n=907 | n=849 | n=211 | n=183 | n=152 | n=134 |
| | | n (%) | n (%) | n (%) | n (%) | n (%) | n (%) | n (%) |
| Age at diagnosis in years | | | | | | | | |
| < 35 | 33 (2.6) | 27 (2.3) | 15 (1.7) | 13 (1.5) | 7 (3.3) | 5 (2.7) | 11 (7.2) | 9 (6.7) |
| 35-50 | 311 (24.5) | 278 (23.8) | 200 (22.1) | 181 (21.3) | 66 (31.3) | 58 (31.7) | 45 (29.6) | 39 (29.1) |
| 51-75 | 741 (58.3) | 679 (58.2) | 553 (61.0) | 518 (61.0) | 114 (54.0) | 96 (52.5) | 74 (48.7) | 65 (48.5) |
| > 75 | 185 (14.6) | 182 (15.6) | 139 (15.3) | 137 (16.1) | 24 (11.4) | 24 (13.1) | 22 (14.5) | 21 (15.7) |
| | | | | | | | | |
| Tumour histology | | | | | | | | |
| ductal (NST) | 1039 (81.8) | 945 (81.0) | 712 (78.5) | 661 (77.9) | 196 (92.9) | 168 (91.8) | 131 (86.2) | 116 (86.6) |
| lobular | 172 (13.5) | 164 (14.1) | 154 (17.0) | 149 (17.6) | 11 (5.2) | 11 (6.0) | 7 (4.6) | 4 (3.0) |
| others | 59 (4.6) | 57 (4.9) | 41 (4.5) | 39 (4.6) | 4 (1.9) | 4 (2.2) | 14 (9.2) | 14 (10.4) |
| | | | | | | | | |
| Tumour size at time of diagnosis | | | | | | | | |
| < 2cm | 636 (50.1) | 603 (51.7) | 511 (56.3) | 486 (57.2) | 79 (37.4) | 73 (39.9) | 46 (30.3) | 44 (32.8) |
| 2-5cm | 528 (41.6) | 479 (41.1) | 340 (37.5) | 317 (37.3) | 104 (49.3) | 89 (48.6) | 84 (55.3) | 73 (54.5) |
| > 5cm | 106 (8.3) | 84 (7.2) | 56 (6.2) | 46 (5.4) | 28 (13.3) | 21 (11.5) | 22 (14.5) | 17 (12.7) |
| | | | | | | | | |
| Nodal status at time of diagnosis | | | | | | | | |
| negative | 780 (61.4) | 722 (61.9) | 584 (64.4) | 529 (64.7) | 119 (56.4) | 105 (57.4) | 77 (50.7) | 68 (50.7) |
| positive | 490 (38.6) | 444 (38.1) | 323 (35.6) | 300 (35.3) | 91 (43.6) | 78 (42.6) | 75 (49.3) | 66 (49.3) |
| | | | | | | | | |
| Tumour differentiation | | | | | | | | |
| G1 | 167 (13.1) | 163 (14.0) | 158 (17.4) | 154 (18.1) | 8 (3.8) | 8 (4.4) | 1 (0.7) | 1 (0.7) |
| G2 | 794 (62.5) | 732 (62.8) | 619 (68.2) | 573 (67.5) | 115 (54.5) | 103 (56.3) | 60 (39.5) | 56 (41.8) |
| G3 | 309 (24.3) | 271 (23.2) | 130 (14.3) | 122 (14.4) | 88 (41.7) | 72 (39.3) | 91 (59.9) | 77 (57.5) |

## Slide 2
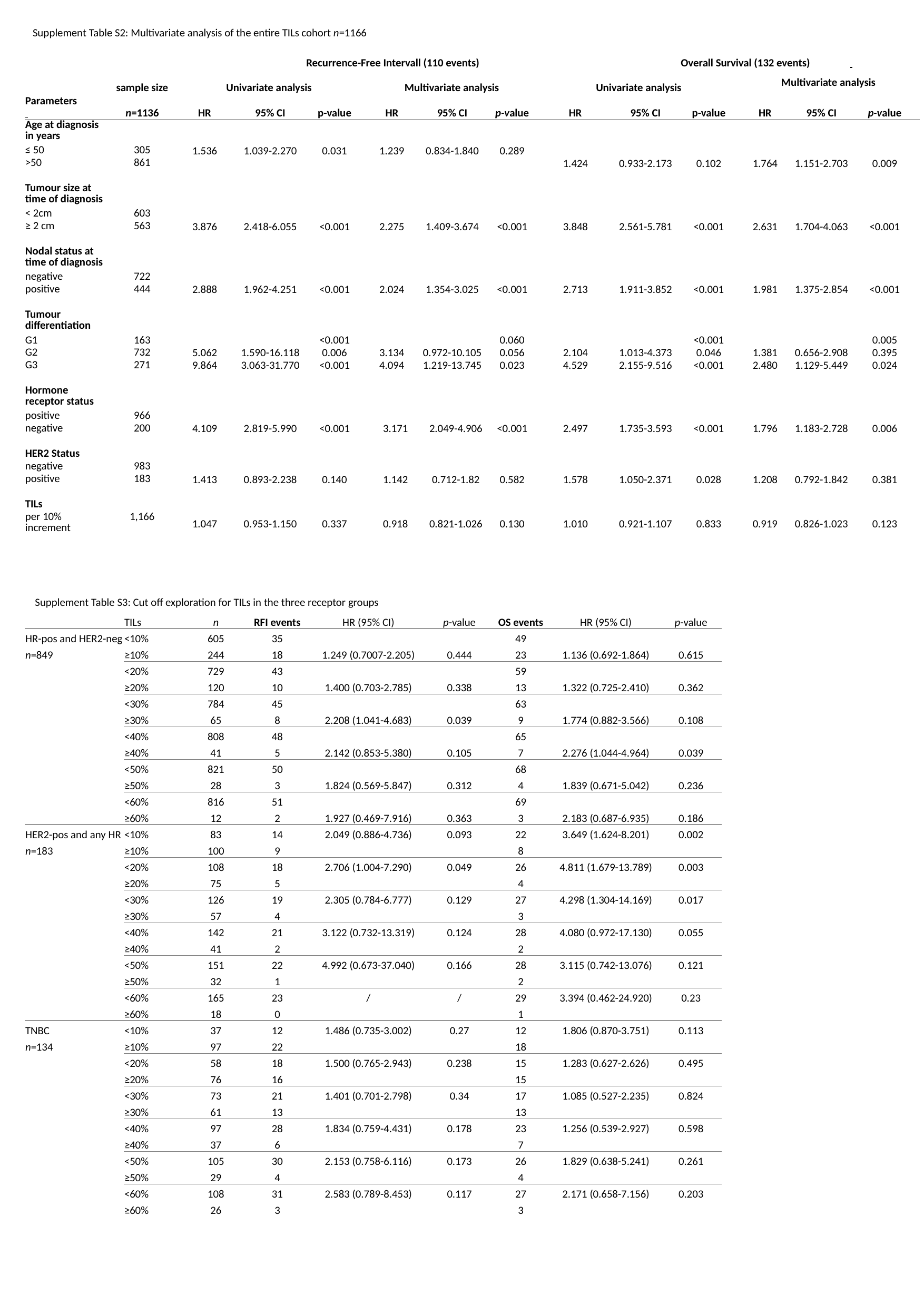

Supplement Table S2: Multivariate analysis of the entire TILs cohort n=1166
| | | | | Recurrence-Free Intervall (110 events) | | | | | | | Overall Survival (132 events) | | | |
| --- | --- | --- | --- | --- | --- | --- | --- | --- | --- | --- | --- | --- | --- | --- |
| Parameters | sample size | Univariate analysis | | | Multivariate analysis | | | | Univariate analysis | | | Multivariate analysis | | |
| | n=1136 | HR | 95% CI | p-value | HR | 95% CI | | p-value | HR | 95% CI | p-value | HR | 95% CI | p-value |
| Age at diagnosis in years | | | | | | | | | | | | | | |
| ≤ 50 | 305 | 1.536 | 1.039-2.270 | 0.031 | 1.239 | 0.834-1.840 | | 0.289 | | | | | | |
| >50 | 861 | | | | | | | | 1.424 | 0.933-2.173 | 0.102 | 1.764 | 1.151-2.703 | 0.009 |
| | | | | | | | | | | | | | | |
| Tumour size at time of diagnosis | | | | | | | | | | | | | | |
| < 2cm | 603 | | | | | | | | | | | | | |
| ≥ 2 cm | 563 | 3.876 | 2.418-6.055 | <0.001 | 2.275 | 1.409-3.674 | | <0.001 | 3.848 | 2.561-5.781 | <0.001 | 2.631 | 1.704-4.063 | <0.001 |
| | | | | | | | | | | | | | | |
| Nodal status at time of diagnosis | | | | | | | | | | | | | | |
| negative | 722 | | | | | | | | | | | | | |
| positive | 444 | 2.888 | 1.962-4.251 | <0.001 | 2.024 | 1.354-3.025 | | <0.001 | 2.713 | 1.911-3.852 | <0.001 | 1.981 | 1.375-2.854 | <0.001 |
| | | | | | | | | | | | | | | |
| Tumour differentiation | | | | | | | | | | | | | | |
| G1 | 163 | | | <0.001 | | | | 0.060 | | | <0.001 | | | 0.005 |
| G2 | 732 | 5.062 | 1.590-16.118 | 0.006 | 3.134 | 0.972-10.105 | | 0.056 | 2.104 | 1.013-4.373 | 0.046 | 1.381 | 0.656-2.908 | 0.395 |
| G3 | 271 | 9.864 | 3.063-31.770 | <0.001 | 4.094 | 1.219-13.745 | | 0.023 | 4.529 | 2.155-9.516 | <0.001 | 2.480 | 1.129-5.449 | 0.024 |
| | | | | | | | | | | | | | | |
| Hormone receptor status | | | | | | | | | | | | | | |
| positive | 966 | | | | | | | | | | | | | |
| negative | 200 | 4.109 | 2.819-5.990 | <0.001 | 3.171 | | 2.049-4.906 | <0.001 | 2.497 | 1.735-3.593 | <0.001 | 1.796 | 1.183-2.728 | 0.006 |
| | | | | | | | | | | | | | | |
| HER2 Status | | | | | | | | | | | | | | |
| negative | 983 | | | | | | | | | | | | | |
| positive | 183 | 1.413 | 0.893-2.238 | 0.140 | 1.142 | | 0.712-1.82 | 0.582 | 1.578 | 1.050-2.371 | 0.028 | 1.208 | 0.792-1.842 | 0.381 |
| | | | | | | | | | | | | | | |
| TILs | | | | | | | | | | | | | | |
| per 10% increment | 1,166 | 1.047 | 0.953-1.150 | 0.337 | 0.918 | | 0.821-1.026 | 0.130 | 1.010 | 0.921-1.107 | 0.833 | 0.919 | 0.826-1.023 | 0.123 |
Supplement Table S3: Cut off exploration for TILs in the three receptor groups
| | TILs | n | RFI events | HR (95% CI) | p-value | OS events | HR (95% CI) | p-value |
| --- | --- | --- | --- | --- | --- | --- | --- | --- |
| HR-pos and HER2-neg | <10% | 605 | 35 | | | 49 | | |
| n=849 | ≥10% | 244 | 18 | 1.249 (0.7007-2.205) | 0.444 | 23 | 1.136 (0.692-1.864) | 0.615 |
| | <20% | 729 | 43 | | | 59 | | |
| | ≥20% | 120 | 10 | 1.400 (0.703-2.785) | 0.338 | 13 | 1.322 (0.725-2.410) | 0.362 |
| | <30% | 784 | 45 | | | 63 | | |
| | ≥30% | 65 | 8 | 2.208 (1.041-4.683) | 0.039 | 9 | 1.774 (0.882-3.566) | 0.108 |
| | <40% | 808 | 48 | | | 65 | | |
| | ≥40% | 41 | 5 | 2.142 (0.853-5.380) | 0.105 | 7 | 2.276 (1.044-4.964) | 0.039 |
| | <50% | 821 | 50 | | | 68 | | |
| | ≥50% | 28 | 3 | 1.824 (0.569-5.847) | 0.312 | 4 | 1.839 (0.671-5.042) | 0.236 |
| | <60% | 816 | 51 | | | 69 | | |
| | ≥60% | 12 | 2 | 1.927 (0.469-7.916) | 0.363 | 3 | 2.183 (0.687-6.935) | 0.186 |
| HER2-pos and any HR | <10% | 83 | 14 | 2.049 (0.886-4.736) | 0.093 | 22 | 3.649 (1.624-8.201) | 0.002 |
| n=183 | ≥10% | 100 | 9 | | | 8 | | |
| | <20% | 108 | 18 | 2.706 (1.004-7.290) | 0.049 | 26 | 4.811 (1.679-13.789) | 0.003 |
| | ≥20% | 75 | 5 | | | 4 | | |
| | <30% | 126 | 19 | 2.305 (0.784-6.777) | 0.129 | 27 | 4.298 (1.304-14.169) | 0.017 |
| | ≥30% | 57 | 4 | | | 3 | | |
| | <40% | 142 | 21 | 3.122 (0.732-13.319) | 0.124 | 28 | 4.080 (0.972-17.130) | 0.055 |
| | ≥40% | 41 | 2 | | | 2 | | |
| | <50% | 151 | 22 | 4.992 (0.673-37.040) | 0.166 | 28 | 3.115 (0.742-13.076) | 0.121 |
| | ≥50% | 32 | 1 | | | 2 | | |
| | <60% | 165 | 23 | / | / | 29 | 3.394 (0.462-24.920) | 0.23 |
| | ≥60% | 18 | 0 | | | 1 | | |
| TNBC | <10% | 37 | 12 | 1.486 (0.735-3.002) | 0.27 | 12 | 1.806 (0.870-3.751) | 0.113 |
| n=134 | ≥10% | 97 | 22 | | | 18 | | |
| | <20% | 58 | 18 | 1.500 (0.765-2.943) | 0.238 | 15 | 1.283 (0.627-2.626) | 0.495 |
| | ≥20% | 76 | 16 | | | 15 | | |
| | <30% | 73 | 21 | 1.401 (0.701-2.798) | 0.34 | 17 | 1.085 (0.527-2.235) | 0.824 |
| | ≥30% | 61 | 13 | | | 13 | | |
| | <40% | 97 | 28 | 1.834 (0.759-4.431) | 0.178 | 23 | 1.256 (0.539-2.927) | 0.598 |
| | ≥40% | 37 | 6 | | | 7 | | |
| | <50% | 105 | 30 | 2.153 (0.758-6.116) | 0.173 | 26 | 1.829 (0.638-5.241) | 0.261 |
| | ≥50% | 29 | 4 | | | 4 | | |
| | <60% | 108 | 31 | 2.583 (0.789-8.453) | 0.117 | 27 | 2.171 (0.658-7.156) | 0.203 |
| | ≥60% | 26 | 3 | | | 3 | | |

## Slide 3
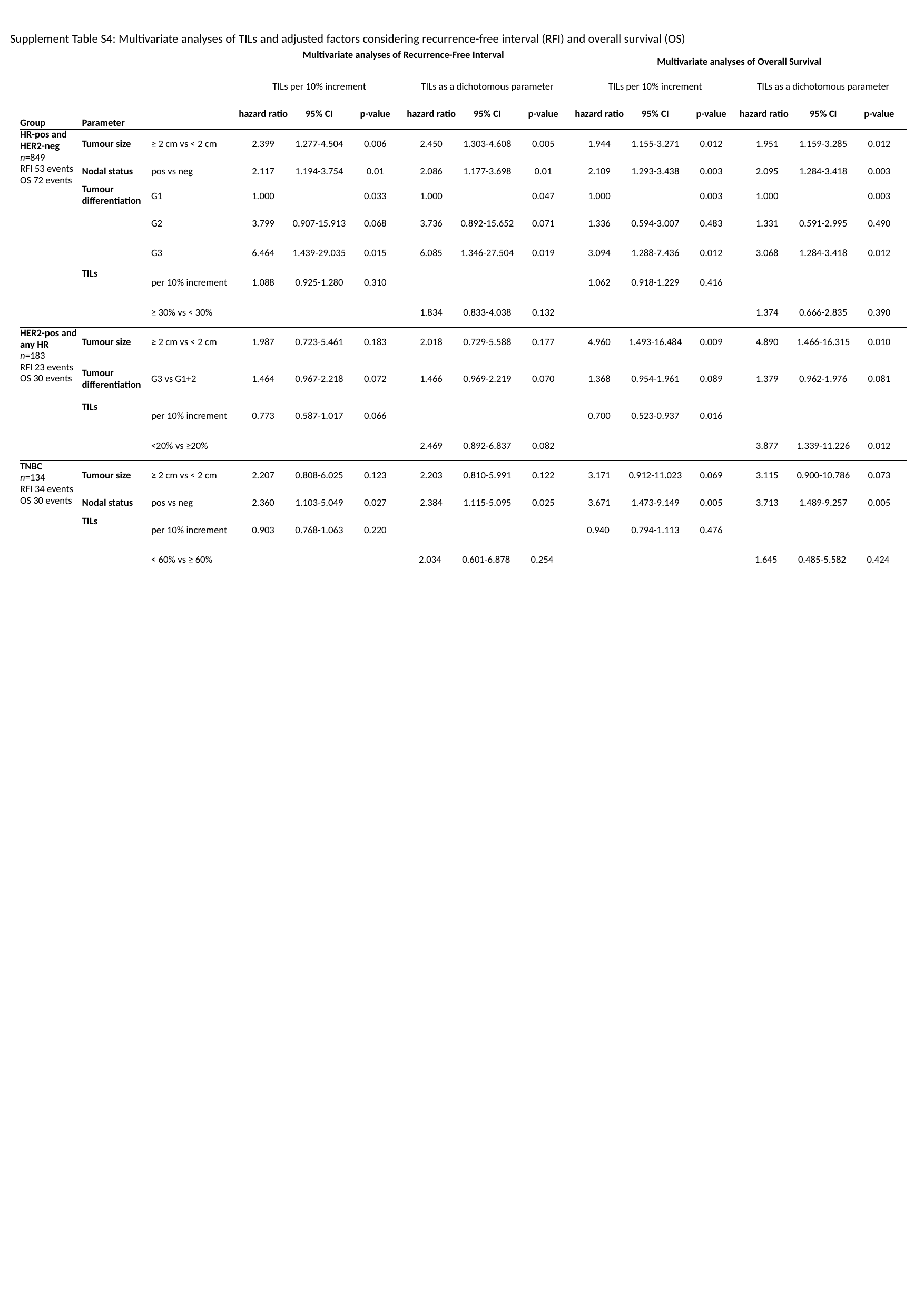

Supplement Table S4: Multivariate analyses of TILs and adjusted factors considering recurrence-free interval (RFI) and overall survival (OS)
| | | | Multivariate analyses of Recurrence-Free Interval | | | | | | Multivariate analyses of Overall Survival | | | | | |
| --- | --- | --- | --- | --- | --- | --- | --- | --- | --- | --- | --- | --- | --- | --- |
| Group | Parameter | | TILs per 10% increment | | | TILs as a dichotomous parameter | | | TILs per 10% increment | | | TILs as a dichotomous parameter | | |
| | | | hazard ratio | 95% CI | p-value | hazard ratio | 95% CI | p-value | hazard ratio | 95% CI | p-value | hazard ratio | 95% CI | p-value |
| HR-pos and HER2-neg n=849 RFI 53 events OS 72 events | Tumour size | ≥ 2 cm vs < 2 cm | 2.399 | 1.277-4.504 | 0.006 | 2.450 | 1.303-4.608 | 0.005 | 1.944 | 1.155-3.271 | 0.012 | 1.951 | 1.159-3.285 | 0.012 |
| | Nodal status | pos vs neg | 2.117 | 1.194-3.754 | 0.01 | 2.086 | 1.177-3.698 | 0.01 | 2.109 | 1.293-3.438 | 0.003 | 2.095 | 1.284-3.418 | 0.003 |
| | Tumour differentiation | G1 | 1.000 | | 0.033 | 1.000 | | 0.047 | 1.000 | | 0.003 | 1.000 | | 0.003 |
| | | G2 | 3.799 | 0.907-15.913 | 0.068 | 3.736 | 0.892-15.652 | 0.071 | 1.336 | 0.594-3.007 | 0.483 | 1.331 | 0.591-2.995 | 0.490 |
| | | G3 | 6.464 | 1.439-29.035 | 0.015 | 6.085 | 1.346-27.504 | 0.019 | 3.094 | 1.288-7.436 | 0.012 | 3.068 | 1.284-3.418 | 0.012 |
| | TILs | per 10% increment | 1.088 | 0.925-1.280 | 0.310 | | | | 1.062 | 0.918-1.229 | 0.416 | | | |
| | | ≥ 30% vs < 30% | | | | 1.834 | 0.833-4.038 | 0.132 | | | | 1.374 | 0.666-2.835 | 0.390 |
| HER2-pos and any HR n=183 RFI 23 events OS 30 events | Tumour size | ≥ 2 cm vs < 2 cm | 1.987 | 0.723-5.461 | 0.183 | 2.018 | 0.729-5.588 | 0.177 | 4.960 | 1.493-16.484 | 0.009 | 4.890 | 1.466-16.315 | 0.010 |
| | Tumour differentiation | G3 vs G1+2 | 1.464 | 0.967-2.218 | 0.072 | 1.466 | 0.969-2.219 | 0.070 | 1.368 | 0.954-1.961 | 0.089 | 1.379 | 0.962-1.976 | 0.081 |
| | TILs | per 10% increment | 0.773 | 0.587-1.017 | 0.066 | | | | 0.700 | 0.523-0.937 | 0.016 | | | |
| | | <20% vs ≥20% | | | | 2.469 | 0.892-6.837 | 0.082 | | | | 3.877 | 1.339-11.226 | 0.012 |
| TNBC n=134 RFI 34 events OS 30 events | Tumour size | ≥ 2 cm vs < 2 cm | 2.207 | 0.808-6.025 | 0.123 | 2.203 | 0.810-5.991 | 0.122 | 3.171 | 0.912-11.023 | 0.069 | 3.115 | 0.900-10.786 | 0.073 |
| | Nodal status | pos vs neg | 2.360 | 1.103-5.049 | 0.027 | 2.384 | 1.115-5.095 | 0.025 | 3.671 | 1.473-9.149 | 0.005 | 3.713 | 1.489-9.257 | 0.005 |
| | TILs | per 10% increment | 0.903 | 0.768-1.063 | 0.220 | | | | 0.940 | 0.794-1.113 | 0.476 | | | |
| | | < 60% vs ≥ 60% | | | | 2.034 | 0.601-6.878 | 0.254 | | | | 1.645 | 0.485-5.582 | 0.424 |

## Slide 4
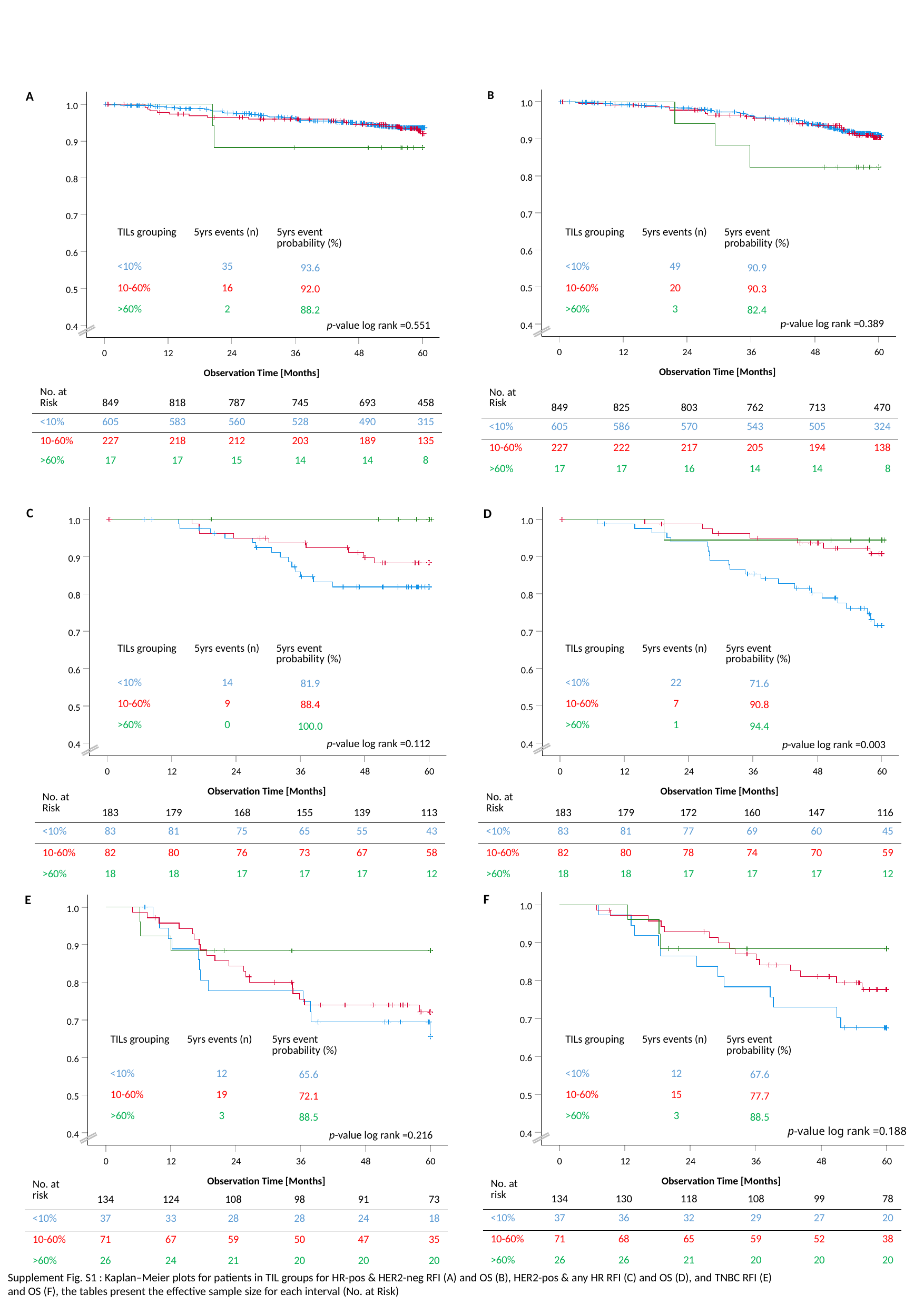

B
A
| TILs grouping | 5yrs events (n) | 5yrs event probability (%) |
| --- | --- | --- |
| <10% | 35 | 93.6 |
| 10-60% | 16 | 92.0 |
| >60% | 2 | 88.2 |
| TILs grouping | 5yrs events (n) | 5yrs event probability (%) |
| --- | --- | --- |
| <10% | 49 | 90.9 |
| 10-60% | 20 | 90.3 |
| >60% | 3 | 82.4 |
| p-value log rank =0.389 |
| --- |
| p-value log rank =0.551 |
| --- |
| No. at Risk | 849 | 818 | 787 | 745 | 693 | 458 |
| --- | --- | --- | --- | --- | --- | --- |
| <10% | 605 | 583 | 560 | 528 | 490 | 315 |
| 10-60% | 227 | 218 | 212 | 203 | 189 | 135 |
| >60% | 17 | 17 | 15 | 14 | 14 | 89 |
| No. at Risk | 849 | 825 | 803 | 762 | 713 | 470 |
| --- | --- | --- | --- | --- | --- | --- |
| <10% | 605 | 586 | 570 | 543 | 505 | 324 |
| 10-60% | 227 | 222 | 217 | 205 | 194 | 138 |
| >60% | 17 | 17 | 16 | 14 | 14 | 8 |
C
D
| TILs grouping | 5yrs events (n) | 5yrs event probability (%) |
| --- | --- | --- |
| <10% | 14 | 81.9 |
| 10-60% | 9 | 88.4 |
| >60% | 0 | 100.0 |
| TILs grouping | 5yrs events (n) | 5yrs event probability (%) |
| --- | --- | --- |
| <10% | 22 | 71.6 |
| 10-60% | 7 | 90.8 |
| >60% | 1 | 94.4 |
| p-value log rank =0.003 |
| --- |
| p-value log rank =0.112 |
| --- |
| No. at Risk | 183 | 179 | 168 | 155 | 139 | 113 |
| --- | --- | --- | --- | --- | --- | --- |
| <10% | 83 | 81 | 75 | 65 | 55 | 43 |
| 10-60% | 82 | 80 | 76 | 73 | 67 | 58 |
| >60% | 18 | 18 | 17 | 17 | 17 | 12 |
| No. at Risk | 183 | 179 | 172 | 160 | 147 | 116 |
| --- | --- | --- | --- | --- | --- | --- |
| <10% | 83 | 81 | 77 | 69 | 60 | 45 |
| 10-60% | 82 | 80 | 78 | 74 | 70 | 59 |
| >60% | 18 | 18 | 17 | 17 | 17 | 12 |
F
E
| TILs grouping | 5yrs events (n) | 5yrs event probability (%) |
| --- | --- | --- |
| <10% | 12 | 65.6 |
| 10-60% | 19 | 72.1 |
| >60% | 3 | 88.5 |
| TILs grouping | 5yrs events (n) | 5yrs event probability (%) |
| --- | --- | --- |
| <10% | 12 | 67.6 |
| 10-60% | 15 | 77.7 |
| >60% | 3 | 88.5 |
| p-value log rank =0.188 |
| --- |
| p-value log rank =0.216 |
| --- |
| No. at risk | 134 | 130 | 118 | 108 | 99 | 78 |
| --- | --- | --- | --- | --- | --- | --- |
| <10% | 37 | 36 | 32 | 29 | 27 | 20 |
| 10-60% | 71 | 68 | 65 | 59 | 52 | 38 |
| >60% | 26 | 26 | 21 | 20 | 20 | 20 |
| No. at risk | 134 | 124 | 108 | 98 | 91 | 73 |
| --- | --- | --- | --- | --- | --- | --- |
| <10% | 37 | 33 | 28 | 28 | 24 | 18 |
| 10-60% | 71 | 67 | 59 | 50 | 47 | 35 |
| >60% | 26 | 24 | 21 | 20 | 20 | 20 |
Supplement Fig. S1 : Kaplan–Meier plots for patients in TIL groups for HR-pos & HER2-neg RFI (A) and OS (B), HER2-pos & any HR RFI (C) and OS (D), and TNBC RFI (E) and OS (F), the tables present the effective sample size for each interval (No. at Risk)

## Slide 5
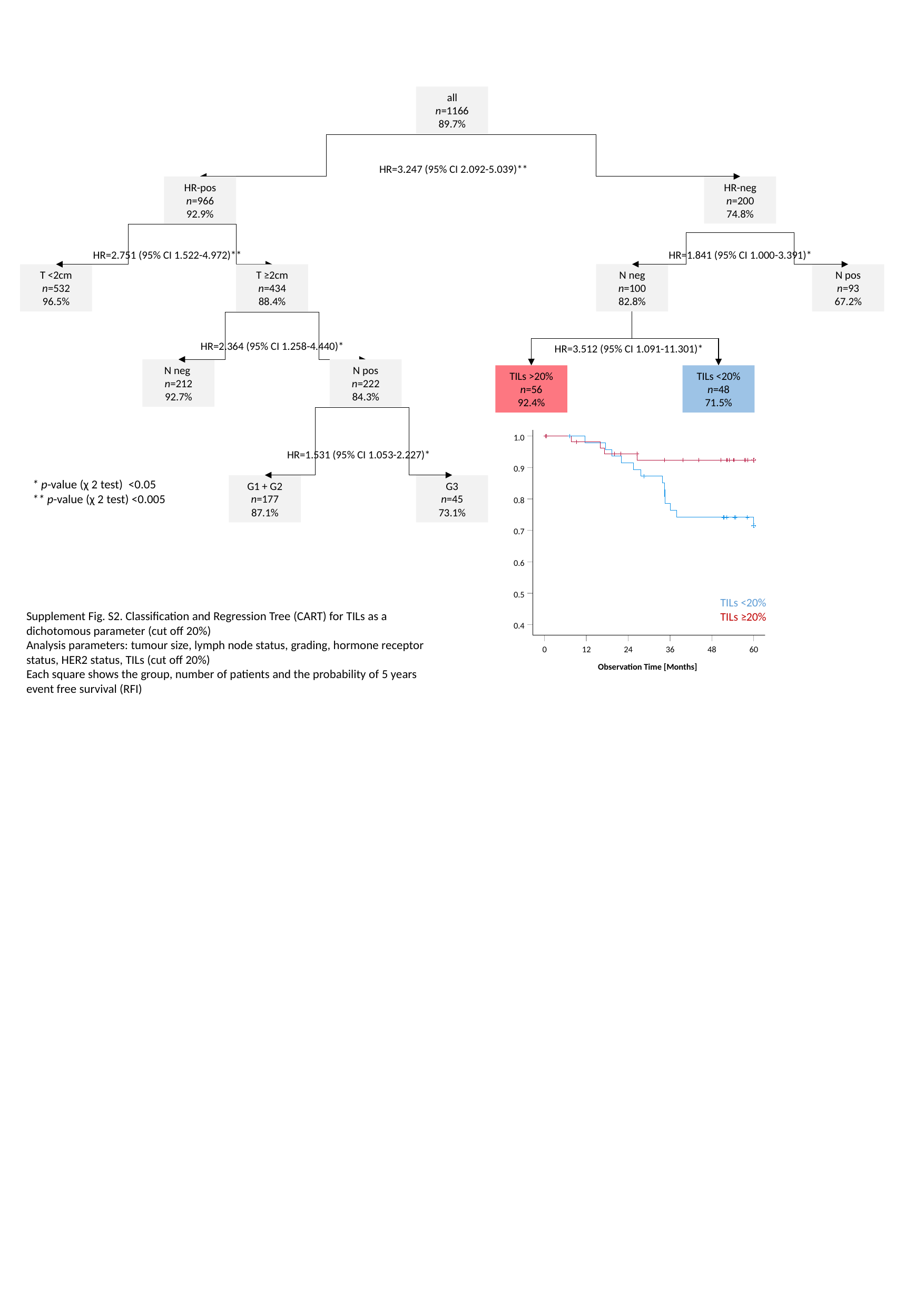

all
n=1166
89.7%
HR-neg
n=200
74.8%
HR=2.751 (95% CI 1.522-4.972)**
N neg
n=100
82.8%
T <2cm
n=532
96.5%
T ≥2cm
n=434
88.4%
N pos
n=93
67.2%
HR=2.364 (95% CI 1.258-4.440)*
N neg
n=212
92.7%
N pos
n=222
84.3%
HR-pos
n=966
92.9%
HR=1.841 (95% CI 1.000-3.391)*
HR=3.247 (95% CI 2.092-5.039)**
HR=1.531 (95% CI 1.053-2.227)*
G1 + G2
n=177
87.1%
G3
n=45
73.1%
HR=3.512 (95% CI 1.091-11.301)*
TILs >20%
n=56
92.4%
TILs <20%
n=48
71.5%
* p-value (χ 2 test) <0.05
** p-value (χ 2 test) <0.005
Supplement Fig. S2. Classification and Regression Tree (CART) for TILs as a dichotomous parameter (cut off 20%)
Analysis parameters: tumour size, lymph node status, grading, hormone receptor status, HER2 status, TILs (cut off 20%)
Each square shows the group, number of patients and the probability of 5 years event free survival (RFI)
TILs <20%
TILs ≥20%
